# Hybrid metamaterials for electrically triggered multifunctional control

**DOI:** 10.1038/ncomms13236

**Published:** 2016-10-27

**Authors:** Liu Liu, Lei Kang, Theresa S. Mayer, Douglas H. Werner

**Affiliations:** 1 Department of Electrical Engineering and Center for Nanoscale Science, The Pennsylvania State University, University Park, Pennsylvania 16802, USA

## Abstract

Despite the exotic material properties that have been demonstrated to date, practical examples of versatile metamaterials remain exceedingly rare. The concept of metadevices has been proposed in the context of hybrid metamaterial composites: systems in which active materials are introduced to advance tunability, switchability and nonlinearity. In contrast to the successful hybridizations seen at lower frequencies, there has been limited exploration into plasmonic and photonic nanostructures due to the lack of available optical materials with non-trivial activity, together with difficulties in regulating responses to external forces in an integrated manner. Here, by presenting a series of proof-of-concept studies on electrically triggered functionalities, we demonstrate a vanadium dioxide integrated photonic metamaterial as a transformative platform for multifunctional control. The proposed hybrid metamaterial integrated with transition materials represents a major step forward by providing a universal approach to creating self-sufficient and highly versatile nanophotonic systems.

## Abstract (web summary)

Despite their exotic properties active metamaterials, where active materials are introduced to advance tunability, switchability and nonlinearity, are seldom reported. Here, Liu *et al*. demonstrate a vanadium dioxide integrated photonic metamaterial as a transformative platform for multifunctional control.

## Graphical abstract

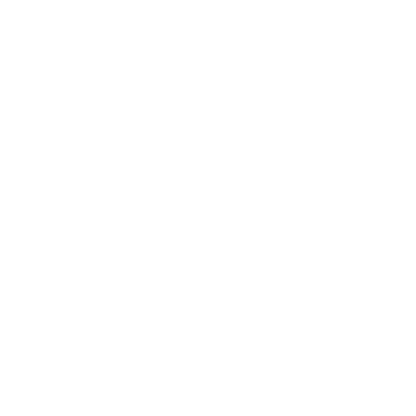

Metamaterials that are able to manipulate electromagnetic radiation in an active manner have been extensively pursued since the emergence of the field^1^,^2^,^3^,^4^,^5^. The resonant properties of conventional metamaterials are derived from their geometric arrangement and material composition^6^,^7^ which are generally fixed. In the optical region, noble metals such as gold (Au) and silver (Ag) possess negative permittivities (that is, Re(*ɛ*)<0) below the plasma frequency and hence are widely adopted to construct subwavelength resonant structural units of photonic metamaterials. In the linear regime, however, the electrical response of metals is negligibly influenced by external fields, offering no avenue for tunability of the metamaterials' response. To mitigate this issue, active media (that is, those possessing variable refractive indices) have been introduced to enable response modulation of various nanostructured systems to an external stimuli. However, compared with the successful hybridizations seen at microwave and terahertz frequencies^8^,^9^,^10^,^11^, explorations have been limited at optical frequencies for the following two main reasons: the lack of available optical materials with non-trivial activity and the difficulties in regulating responses to external forces in an integrated manner. It is also worth noting that compared with integrated photonic circuits, such as waveguide ring resonators, whose optical modes are able to be switched by small waveguide index changes arising from their high *Q* resonance^12^, metal-dielectric photonic metamaterials usually require non-trivial index variations to achieve observable activity.

Consequently, in optics, various approaches to the realization of active nanophotonic systems have been explored and can be roughly classified by the means to which the refractive index can be tuned, including from the intrinsic birefringence of materials that exhibit a large electro-optic coefficient, for example, liquid crystals^13^,^14^,^15^, and ferroelectric materials such as barium titanate (BaTiO_3_)^16^, from the carrier excitations in semiconductors such as Silicon^17^,^18^, quantum dots such as CdSe quantum dots^19^, and graphene^20^,^21^ as well as from the transition of phase change materials^22^,^23^,^24^,^25^,^26^,^27^,^28^,^29^,^30^,^31^,^32^,^33^,^34^,^35^,^36^,^37^. Moreover, in accordance with the Clausius–Mossotti relation, the change in refractive index arises by varying the polarizability *α*, albeit by different mechanisms. Materials that possess large electro-optic coefficients achieve their large birefringence properties via the intrinsic anisotropy resulting from reorientation of molecules (or ferroelectric domains) under application of external fields. Polarizability changes realized via carrier excitations, however, often require extreme conditions, for example, intensive optical pumping. Whereas, in sharp contrast, transitions in phase change materials, which deform the lattice structure, would lead to drastic variations in *α*, and thus the refractive index, over a broad spectral range. In light of this, vanadium dioxide (VO_2_), a classical transition metal oxide that behaves as an insulator (monoclinic phase) and half-filled metal (3*d*^1^, (*S*=1/2), tetragonal phase) at temperatures below and above the insulator-to-metal transition (IMT) temperature (*T*_IMT_∼67 °C in bulk crystals)^38^, respectively, has been used to facilitate tunable metamaterials. In these systems the IMT of VO_2_ has been achieved through either direct thermal control^23^,^24^,^25^,^26^,^27^,^28^,^31^,^32^,^33^,^34^ or by the resonance-enhanced opto-thermal effect^30^,^35^. A detailed analysis of the VO_2_-based metadevices that have been reported on to date can be found in Supplementary Table 1 and Supplementary Note 6. Interestingly, temperature-tuned infrared radiation with negative differential thermal emittance has been reported recently in a VO_2_ film, which, in a sense, acts as a natural metamaterial^32^.

Electrically controlled active metamaterials are an essential component for the successful realization of metadevices, which are seen to be the next frontier with great importance in a wide range of practical applications. Despite controversy over their microscopic origins, current and field-effect-induced phase transitions in VO_2_ have been reported^39^,^40^, which suggests that VO_2_ is a promising candidate as an active medium for the creation of electrically modulated hybrid metamaterials. However, a close inspection of these studies reveals that the observed transition is extremely localized and hinges on electrical current filaments, suggesting that this direct electrically induced transition may not be sufficient to enable detectable modulations in metamaterials. Recently, nano-engineered metals have been comprehensively used in metamaterial and plasmonic systems, because in addition to facilitating optical resonances their electrical conductivities enable complex functionalities for achieving plasmonic-enhanced electro-optic effects in both the linear and nonlinear regimes^41^,^42^,^43^. Metadevices with intrinsically embedded electrical and optical functions are correspondingly referred to as ‘self-sufficient' or ‘self-contained'. Beyond that, metals are also thermally conductive, a property that could be exploited to achieve exotic metadevices based on the electro-thermal effect.

Here, by integrating a VO_2_ film of nanoscale thickness into an optical metamaterial absorber, we experimentally demonstrate a hybrid metamaterial platform that achieves electrically triggered multifunctional control in the mid-infrared region. The proposed self-sufficient and highly versatile metamaterial system embodies a direct monolithic integration of phase transition materials into photonic structures. Acting as part of the resonating structure, VO_2_ enables the tunable nature of the device, whereas the nano-engineered metals simultaneously support the optical resonances and the electrical control of the phase transition triggered through a Joule heating effect.

## Results

### An electrically active metamaterial based on IMT

**Figure 1:**
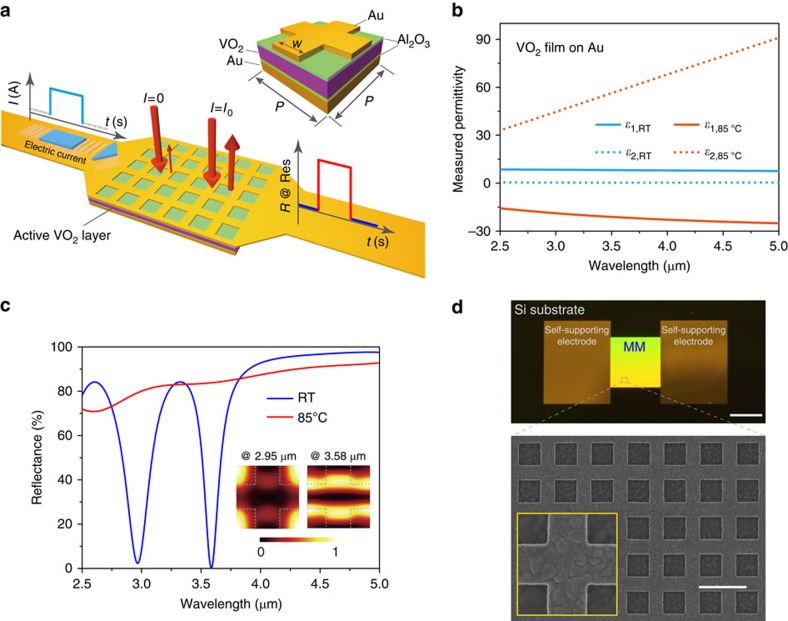
IMT enabled electrically actuated metadevice. (**a**) Three-dimensional illustration of the metamaterial device consisting of a sandwich system with a 100 nm-thick patterned-mesh top gold (Au) layer, a 260 nm-thick active VO_2_ layer and an optically thick (200 nm) Au ground plane. A 50 nm-thick Al_2_O_3_ layer is applied in between both gold/VO_2_ interfaces for optimized device performance. Unit cell of the metamaterial absorber is depicted in the inset where *P*=1550, nm and *w*=600 nm. The top patterned gold layer is connected to an external circuit, which simultaneously supports optical resonances and electrical functionality for Joule heating. The reflection of incident light at normal incidence would be immediately tuned as a function of the electrical current flowing through the layer, which triggers the IMT in the active VO_2_ layer. (**b**) Measured permittivity of VO_2_ thin film on top of Au at RT and 85 °C, which clearly indicates the phase transition. (**c**) Simulated reflectance spectra of the hybrid metamaterial absorber with VO_2_ layer in the insulator (blue curve) and metallic (red curve) phases. Insets: calculated electric field profiles (|*E*|^2^) within the VO_2_ layer for a unit cell (inset of **a**) at the wavelengths of 2.95 and 3.58 μm. (**d**) Photo of the device and scanning electron microscope (SEM) image of the metadevice, with the inset illustrating an enlarged SEM image of a unit cell. The scale bars in the upper and lower panel of **d** represent 2 mm and 1 μm, respectively.

Figure 1a shows a schematic of the hybrid metamaterial absorber that consists of two continuous metallic layers sandwiching an active VO_2_ thin film instead of the inactive dielectric used in typical configurations^44^. In our design, in addition to supporting the resonant absorption modes, the top mesh-patterned Au layer is extended and connected to an external circuit, which, simultaneously, allows Joule heating as a result of the in-plane current flow. Furthermore, the high thermal conductivity of Au facilitates Joule heat conduction into the VO_2_ thin films, leading to a thermally induced IMT. Thus, the reflection characteristics at the resonances can be tuned as a function of the applied electrical current. It is noteworthy that the reflectance, transmittance and absorption properties of the metamaterials satisfy the relation *R*(*ω*)=1−*A*(*ω*) and *T*(*ω*)=0. To optimize the design, we began by measuring the temperature-dependent dielectric permittivity (*ɛ*=*ɛ*_1_+*iɛ*_2_) of a VO_2_ film grown on Au, rather than on the typical sapphire substrate (see Methods). As illustrated in Fig. 1b, the VO_2_ thin film behaves similar to a low-loss dielectric at room temperature (RT) but a Drude-like metal at 85 °C, indicating the thermally induced phase transition. Then, using the measured dielectric properties of VO_2_ (Fig. 1b), we simulate the reflection of the hybrid metamaterial unit cell at normal incidence. A thin (50 nm) Al_2_O_3_ spacer is introduced at both VO_2_ and Au interfaces for two reasons: first, to lower the impact of the loss of VO_2_ at RT to achieve sharper resonant absorption modes and, second, to serve as a diffusion barrier that allows crystallization of VO_2_. In contrast to past tunable metamaterial embodiments that have relied on active materials serving as bulk substrates, our design leverages the photonic enhancement to the greatest extent by incorporating the subwavelength-thick VO_2_ thin-film (∼260 nm that <*λ*_res_/12) directly into the resonating structure (see electric field profiles depicted in the inset of Fig. 1c). This could become a viable path forward for the creation of highly sensitive and ultra-compact electro-optic devices. As illustrated in Fig. 1c, a drastic variation of reflection takes place around the wavelengths of 2.95 and 3.58 μm, that is, the two absorber modes of the metamaterial at RT, respectively. Figure 1d depicts a photograph and scanning electron microscopic images of the fabricated device that is electrically functionalized via on-chip connections. We note that in spite of a variety of VO_2_ thin-film preparation procedures such as molecular beam epitaxy, pulsed laser deposition and sputtering, growing a thick single-phase VO_2_ layer on a metal substrate remains a challenge. In this study, mediated by an atomic layer deposition (ALD) enabled Al_2_O_3_ barrier layer, the single-phase poly-crystalline VO_2_ thin film is achieved with a two-step sputter/anneal process on top of the Au ground plane (see Supplementary Note 1 and Supplementary Fig. 1).

**Figure 2:**
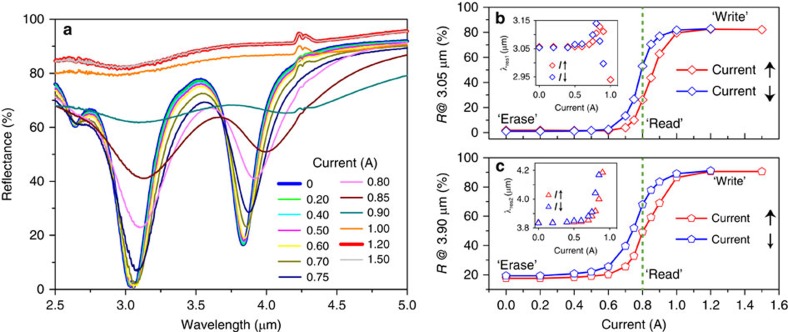
Electrically tuning the spectral response of hybrid metamaterials. (**a**) Measured reflection spectra for various intensities of electrical current applied. A drastic but continuous spectrum tuning is achieved before saturation. (**b**,**c**) Hysteresis in the on-resonance reflectance, which is dependent on the applied electrical current (*I*), with a sweeping rate of 0.1 A min^−1^. The corresponding resonance wavelength (*λ*_res_) as a function of *I* is shown in the inset.

By collecting reflection spectra with a Fourier transform infrared (FTIR) spectrometer (see Supplementary Note 2 and Supplementary Fig. 2) at different electrical current (*I*) values applied to the top mesh-patterned Au layer (Fig. 1a), we first determined the electrical tuning of the metamaterial optical response in thermal equilibrium, as illustrated in Fig. 2a. In particular, relative to the static reflectance (when *I*=0 A), absolute tunings of 80% and 75%, which correspond to 75-fold and 5-fold relative modulations (defined as 
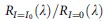
), are observed at wavelengths of 3.05 and 3.85 μm, respectively, when *I* increases to 1.20 A, which is in good agreement with the simulation data (Fig. 1c). For in-between values of *I*, a continuous tuning is observed. This gradual change can be attributed to the percolation progress^30^, which produces localized phase changes that alter the effective permittivity of VO_2_ and thus the response of the metamaterial. This is further verified by the spectroscopic results obtained by directly controlling the device temperature with the external heating system (see Supplementary Note 3 and Supplementary Fig. 3). Furthermore, collecting data for a cyclic change of the electrical current, in Fig. 2b we illustrate the on-resonance reflectance as a function of increasing and then decreasing *I*. It can be seen that the modulation becomes obvious only when the intensity of *I* exceeds ∼0.5 A, a threshold subject to the overall thermal condition of the device, and saturates when the intensity of *I* exceeds 1.0 A. Furthermore, a hysteresis behaviour is seen and reaches its maximum value at *I*=0.8 A where the slope of the current-dependent reflectance is the steepest. In addition, the resonance frequencies of our hybrid metamaterials are also regulated, as shown in the insets of Fig. 2b,c. We note that Fig. 2 not only illustrates the mechanism of the tuning behaviour, but it also provides the basis for constructing a self-sufficient platform that supports electrically triggered multifunctional control. To highlight the versatility of the proposed metadevice for multifunction control, hereinafter we present a series of proof-of-concept demonstrations.

### Electrically switchable reflection

**Figure 3:**
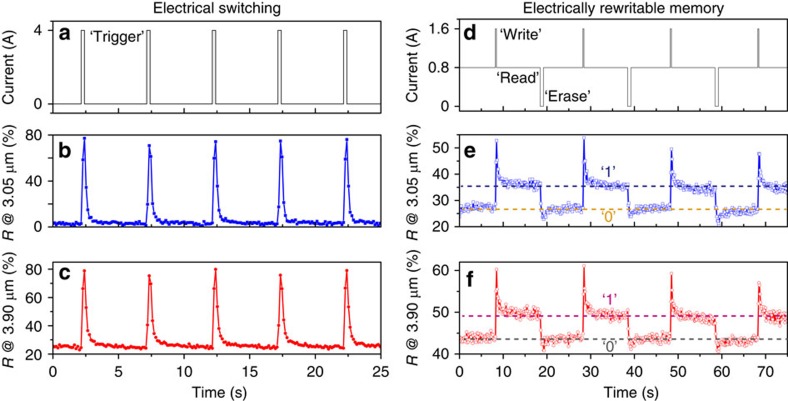
Reflectance switching and rewritable memory effect observed. (**a**–**c**) Observation of electrical switching of reflectance at the two resonant modes. Electrical current pulses with a width of 0.25 s are used to ‘trigger' localized MIT in the VO_2_ film, which enables time-resolved reflectance of the metadevice at a sub-second time scale. (See Supplementary Fig. 3 for the dependence of the switching operation on the electrical current intensity). (**d**–**f**) Electrically rewritable memory effect observed in the metadevice. As the vertical dashed lines in Fig. 2b,c indicated, a bias current of 0.8 A was applied to achieve the maximum hysteresis. On top of that, an additional 0.25 s (0.75 s) current pulse of 0.8 A (−0.8 A) is used to ‘write' (‘erase') the rewritable photonic memory in the reflection signals.

A closer examination of the results shown in Fig. 2b immediately reveals that two types of electro-optic functionality are enabled by the electrical current-dependent reflectance, that is, reversible reflection switching, obtained by applying *I* at low (or zero) and saturated levels, as well as the memory effect due to hysteresis. By applying a series of electrical current pulses, while recording the magnitude of the reflectance (as shown in Fig. 3a–c), we experimentally demonstrated the electrical switching effect. When a rectangular-shaped current pulse is applied, a remarkable amplitude switching capability as large as 75% and 55% of absolute tuning is observed at the two resonances, respectively, as shown in Fig. 3b,c. The slight fluctuation of the maximum tuning magnitude is attributed to the limited time-sampling rate (10 Hz) of our FTIR spectroscopy system. Besides the saturating current observed in Fig. 2b, a ‘trigger' was implemented by applying a 4.0 A-high and 0.25 s-wide electrical current pulse train (Fig. 3a) to improve the dynamic response due to the fact that, under certain thermal conditions, VO_2_ experiences faster phase transitions with higher input Joule heating powers (see Supplementary Note 4 and Supplementary Fig. 4). The measured results indicate a rising and falling edge of ∼0.2 and ∼0.5 s, respectively, in the time trace of on-resonance reflectance. It is postulated that finer thermal engineering, which reduces the thermal capacity of the device, may yield even better dynamic performance.

### Electrically erasable and programmable memory effect

By exploiting the intrinsic hysteresis behaviour of VO_2_, an electrically controlled memory can be realized. To switch between the ‘0' and ‘1' states, positive (0.8 A, 0.25 s) and negative (−0.8 A, 0.75 s) current pulses referred as the ‘write' and ‘erase' inputs of this memory process, respectively, are applied in a circular sequence on top of a 0.8 A bias current which maintains the VO_2_ at its maximum hysteresis point indicated in Fig. 2b,c. The measured results in Fig. 3e,f reveal a flip-flop operation in reflectance, unambiguously manifesting as an electrically erasable and programmable read-only memory effect. In particular, as indicated by the dashed lines, the ‘1' and ‘0' states offer absolute reflectance contrasts of ∼10 and ∼5% at the resonant wavelengths for given experimental conditions, allowing us to read the programmed reflectance out as long as the information is stored. The temperature fluctuations of the device arising from environment temperature changes were kept within 0.3 °C during the measurements. We emphasize that, compared with the previously reported memory effect in metamaterials^22^,^45^, the proposed photonic metadevice demonstrated here successfully achieves at least three major competitive advantages: biasing without an external temperature control, operating in the mid-infrared wavelength region and exhibiting a dual-wavelength clear binary contrast for storing and reading information. Moreover, considering the planar and compact configuration of the metamaterial, the above modulations can be conducted in an energy-efficient manner. For instance, the input power level in our switching measurements was ∼1 μJ per unit cell per pulse and may be able to be further reduced. It is also worth noting that the transition temperature of VO_2_ can be tailored to RT by various materials engineering approaches^32^, which would potentially eliminate the bias requirement and facilitate non-volatile memory applications.

### Tuning the spatial dependence of infrared images

**Figure 4:**
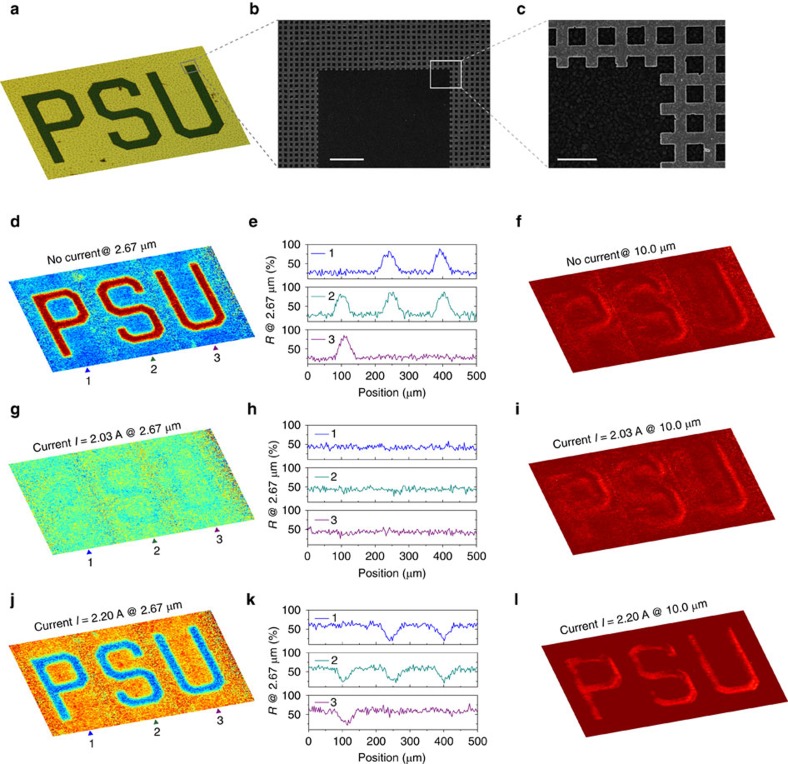
Electrically controlling spatially dependent infrared images. (**a**) Microscopic image of the fabricated metamaterials with an area of the top Au layer removed to form the letters ‘PSU'. Geometrical dimensions of imaging: 900 × 500 μm^2^. (**b**,**c**) Scanning electron microscope (SEM) images of a corner of the patterned letter ‘U'. The top gold meshes, which are connected to external circuitry, correspond to the olive area in **a**, whereas bare VO_2_ forms the letters in dark green in **a**,**d**,**g**,**j**, infrared images at a wavelength of 2.67 μm of the metamaterial with various electrical currents applied. Interestingly, as illustrated in **g**, the letters became invisible in the infrared image at a proper electrical current intensity. (**e**,**h**,**k**) The corresponding reflectance distribution across each of the letters at locations labelled by ‘1', ‘2' and ‘3'. (**f**,**i**,**l**) Infrared images of the device at wavelength of 10.0 μm. No obvious image tuning effect is observed. Scale bars, 10 and 2 μm (**b**,**c**).

In addition to the spatially selective absorptivity previously demonstrated with a static metamaterial absorber, the proposed electrically active metadevice can enable even more sophisticated spatial modulation schemes of infrared signals. To demonstrate this versatility, as indicated by the optical microscope and scanning electron microscope images illustrated in Fig. 4a–c, we fabricated the same structure as was used for the measurements discussed in Fig. 2, except that a portion of the top mesh-patterned Au layer was removed to form three letters, ‘PSU'. Again, the top Au layer was extended and connected to an external circuit (not shown) for achieving an electrical control. The measured two-dimensional infrared images reveal that when *I*=0 A (Fig. 4d) the letters of ‘PSU' stand out with high reflectance, compared with the low-reflective regions of the metamaterial. Very interestingly, with an applied current of *I*=2.03 A, the ‘PSU' text faded into the background of the infrared image (Fig. 4g) and became invisible. Moreover, when *I* was further increased, the ‘PSU' image was observed as an intaglio on a highly reflective background (Fig. 4j). Plotting the spatial distribution of reflectance across each of the letters, Fig. 4e,h,k quantitatively indicates the infrared display contrast in these three scenarios. On the contrary, the infrared image of the sample at the off-resonance wavelengths is not closely correlated with the electrical current applied. We note that the off-normal incidence effect causes deviation of the spectroscopic response during the imaging (with effective numerical aperture (NA)=0.5) from the results shown in Fig. 2 (see Supplementary Fig. 5 for details). In addition, the reflection from the letters decreases with applied current (temperature) due to variations in the dielectric constant of VO_2_^32^, which actually introduces an additional degree of freedom for spatial modulation engineering.

## Discussion

The temporal performance of metadevices is of importance for a number of potential applications. VO_2_-based hybrid metamaterials have the potential for ultrafast modulation and switching due to the fact that subpicosecond response times of VO_2_ have been observed in different systems involving thermal processes^30^,^46^. However, the switching speed of the proposed metadevice is limited by the thermal and input-power parameters at the device level rather than the intrinsic limit of VO_2_. Nevertheless, its switching speed can be dramatically improved through primarily localized Joule heating and by lowering the volumetric heat capacity of the entire device. To qualitatively illustrate the operation of the metadevice, we performed a series of electrical-thermal simulations using COMSOL Multiphysics software package (see Supplementary Fig. 6). The simulations reveal that applying short but high-intensity current pulses and/or reducing the device dimensions can significantly increase the switching speed without sacrificing the modulation depth (see Supplementary Note 5 and Supplementary Figs 7 and 8 for details). In addition to device-level thermal engineering, better crystallization control of VO_2_ and re-optimization of the thickness of the low thermal conductivity buffer layer (Al_2_O_3_) should also improve the temporal performance of the proposed metadevice.

By virtue of Kirchhoff's law, emissivity of a thermal emitter at equilibrium should be equal to its absorptivity^47^. Therefore, the metamaterial can also serve as an infrared emitter with spatial and temporal emissivity that can be purposely controlled via electrical current. In this regard, we note that from the point of view of thermal detectors, the metadevice, counterintuitively, will look cooler when electrically heated due to negative differential thermal on-resonance emittance^32^ as indicated by the results in Fig. 2. According to Wien's displacement law, the peak-radiated power produced by a black body occurs at a wavelength *λ*_max_=*b*/*T*, where *b* is Wien's displacement constant and *T* is the corresponding temperature. However, the temperature range 20–80 °C corresponds to peak radiation efficiencies around 8 and 10 μm, respectively, which are well outside the wavelength of interest in this study. In other words, the hybrid metamaterial would be a very inefficient thermal emitter. Luckily, the VO_2_-based hybrid metamaterial could be optimized to operate at longer wavelengths, for example, 5–15 μm, where the thermal radiation efficiency is much higher. Alternatively, controllable thermal emission in the near- and mid-infrared regions can be realized through hybrid metamaterials integrated with other transition materials such as NbO_2_ whose transition occurs near 800 °C (ref. 48), corresponding to *λ*_max_≈2.6 μm. The ability to tune metamaterials through their geometries, as well as the integration capability demonstrated in this work offer unprecedented flexibility for realizing thermal emitters under the design of task paradigm. A detailed study of this phenomenon may reveal many desirable applications such as active camouflage via thermal chameleon coatings, but is beyond the scope of this work.

Finally, it is worth providing an overview of the progress of VO_2_ metadevices by making a comparison between our metadevice and those reported in the literature. As illustrated in Supplementary Table 1 and Supplementary Note 6, there are few electrically actuated VO_2_ metadevices operating in the mid- and near-infrared regimes (with the majority of the reported cases being designed to operate in the THz regime). In addition, regarding the temporal performance, studies of device functionality based on the use of a heating stage have indicated slow reaction times due to a reliance on global temperature changes^23^,^24^,^25^,^26^,^27^,^28^,^31^,^32^,^33^,^34^. Nevertheless, ultrafast modulation arising from optically driven transition has also been observed^30^. Furthermore, most of the reported studies introduced VO_2_ into the hybrid systems as a part of the substrate, which, to some extent, limits the potential modulation depth and temporal response characteristics of the metadevice. In contrast to the reported studies, operating at a wavelength around 3 μm, our metadevice required employing nanoscale fabrication (VO_2_ thin film growth on metallic substrates) and a highly advanced comprehensive electrical/optical control system. More importantly, the proposed metamaterial formed by topologically continuous plasmonic structures simultaneously enables both perfect absorption and the application of electrical signals across the structure. To the best of our knowledge, this design concept, which enables ‘self-contained' or ‘self-sufficient' active plasmonic devices, has not previously been implemented for IMT-based metadevices. Moreover, in our design the VO_2_ thin film acts as part of the resonating structure, which not only ensures a large modulation depth but also offers the potential for dynamic switching solely involving localized thermal processes. Therefore, beyond being technologically distinct from previously reported studies, this core design concept provides a universal approach to generating self-sufficient and highly versatile nanophotonic systems.

In summary, this work demonstrates that electrified metamaterial absorbers integrated with vanadium dioxide could be used as self-sufficient versatile metadevices whose temporal, frequency and spatial reflection characteristics can be purposely controlled by an applied electrical current. The unification of conventional and plasmonic properties of metals with the phase transition of naturally active materials is expected to establish a new paradigm in metamaterial-based multifunctional systems for electrically triggered information processing, storage and display. We believe that the unit-cell nature of metamaterials and the active tuning capability demonstrated here can be combined to provide unprecedented flexibility for pixelated light manipulation in the subwavelength regime. We also envision that the intriguing interplay between photonic nanostructures and the recently developed active materials family of two-dimensional transition-metal dichalcogenides would bring forth exciting physics and novel functionalities in the realization of atomic-scale photonics.

## Methods

### Numerical simulations

Full-wave electromagnetic simulations were performed using CST Microwave Studio, a commercial finite integration package. A unit cell of the investigated structure is simulated using periodic boundary conditions. The material parameters of VO_2_ measured at RT and 85 °C are employed in the simulations to investigate the tuning response of the hybrid metamaterials arising from the IMT of VO_2_.

### Device fabrication

The hybrid metamaterial (that is, metadevice) was fabricated on a silicon substrate. The SiO_2_/Au/Al_2_O_3_ layer stack was formed on top of the Si substrate by thermal oxidation/e-gun evaporation/ALD. A VO_*x*_ (*x*<2) film was deposited on top of Al_2_O_3_ using pulsed DC reactive ion sputtering and then further oxidized and crystallized by thermal annealing to form single-phase VO_2_. After that, the sample was coated with a second layer of Al_2_O_3_ with the same ALD process and then the Au mesh was defined on top of the second Al_2_O_3_ layer with e-beam lithography and a lift-off process. A detailed description of the fabrication process flow can be found in Supplementary Note 1.

### Optical characterization

The reflectance of the hybrid metamaterial absorber was characterized using FTIR spectroscopy. A customized accessary was employed with a Bruker IFS 66/s spectrometer to realize the normal reflectance measurement. For the results shown in Figs 2 and 3, the hybrid metamaterial sample was placed on a microscope slide, which was then mounted on the sample holder in the customized FTIR accessary. To enable electrically controlled modulation, the top nano-patterned Au mesh layer (Fig. 1d) was connected to external circuitry with the input current waveforms programmed by a function generator and an amplifier. To monitor the temperature fluctuation during the memory effect measurements, a thermal couple was placed 1 mm above the surface of the metadevice. To acquire the spatially dependent infrared images shown in Fig. 4, the hybrid metamaterial sample was photographed using a 128 × 128 focal plane array detector integrated in the hyperion 3,000 microscope of the FTIR system. The objective lens of the microscope was a 15 × condenser with an average incidence and collection angle of light ∼30°.

### Data availability

The data that support the findings of this study are available from the corresponding author on request.

## Additional information

**How to cite this article:** Liu, L. *et al*. Hybrid metamaterials for electrically triggered multifunctional control. *Nat. Commun.* **7,** 13236 doi: 10.1038/ncomms13236 (2016).

**Publisher's note:** Springer Nature remains neutral with regard to jurisdictional claims in published maps and institutional affiliations.

## Supplementary Material

Supplementary Information Supplementary Figures 1-8, Supplementary Table 1, Supplementary Notes 1-6 and Supplementary References.
