# Supplementary material for: Hybrid metamaterials for electrically triggered multifunctional control
Source: Nat Commun. 2016 Oct 27;7:13236. doi: 10.1038/ncomms13236 (PMC5095288; doi:10.1038/ncomms13236)
Supplement: Supplementary Information — Supplementary Figures 1-8, Supplementary Table 1, Supplementary Notes 1-6 and Supplementary References. [file ncomms13236-s1.pdf]

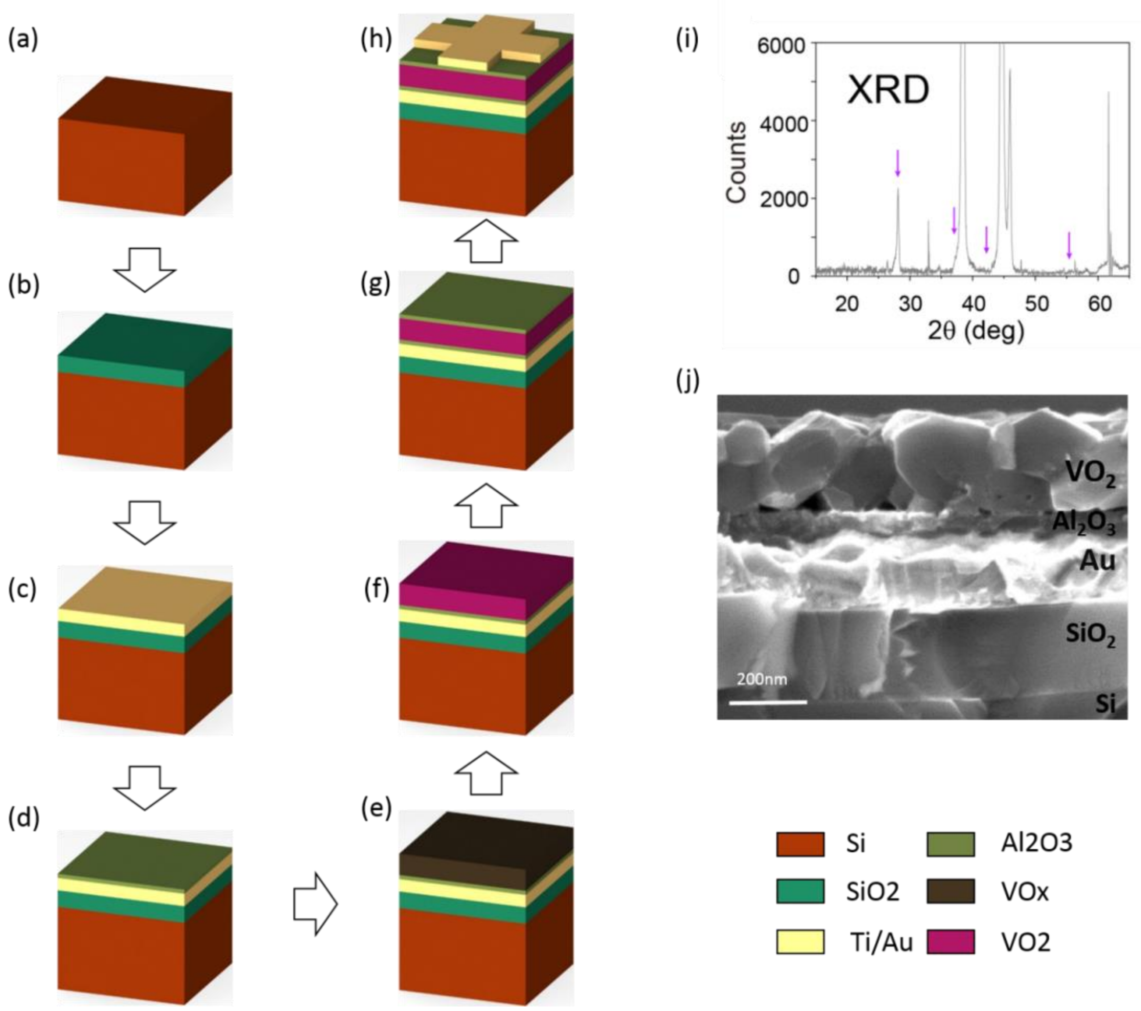

Supplementary Figure 1. Device fabrication. (a-h) Fabrication process flow of the VO_2 metamaterial absorber. (i) XRD spectrum of the VO_2 film after step f. (j) Cross-sectional SEM image of the film stack after step f.

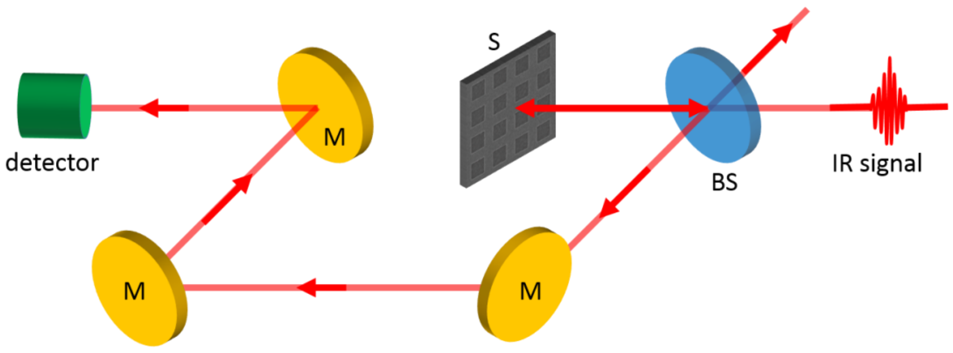

Supplementary Figure 2. Light path of the normal reflectance accessary. Labels of each component are listed as following: M-- mirror; S-- sample; BS-- beam splitter.

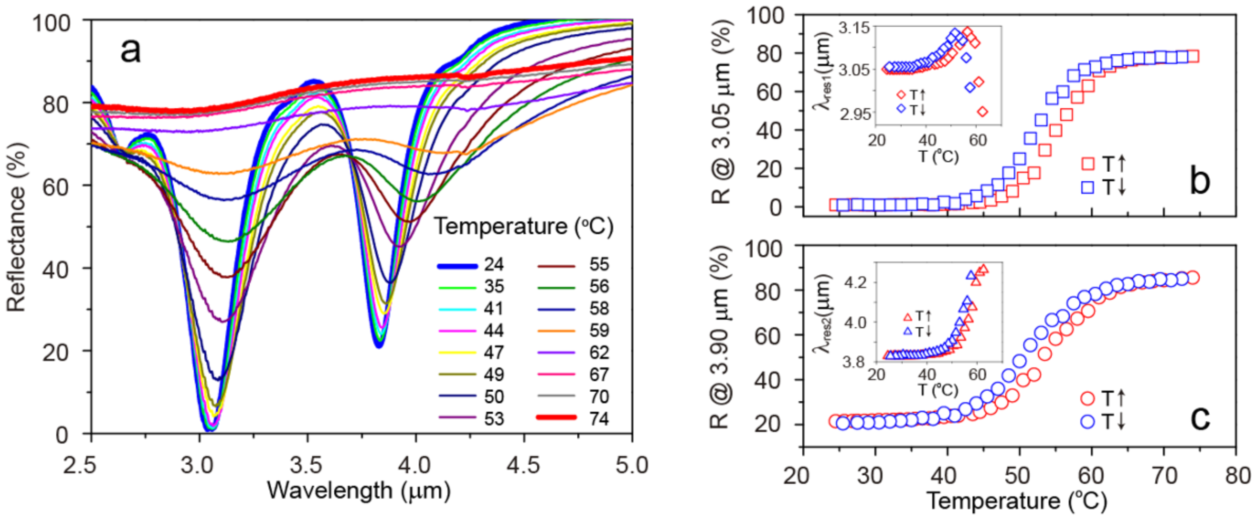

Supplementary Figure 3. Thermally tuning the spectral response of the hybrid metamaterial. **a**, Measured reflection spectra for various sample temperatures. **b-c**, Hysteresis dependence of the on-resonance reflectance on the temperature with a sweeping rate of 2 °C min^{-1}. The corresponding resonance wavelength (λ_{res}) as a function of the temperature is shown in the inset.

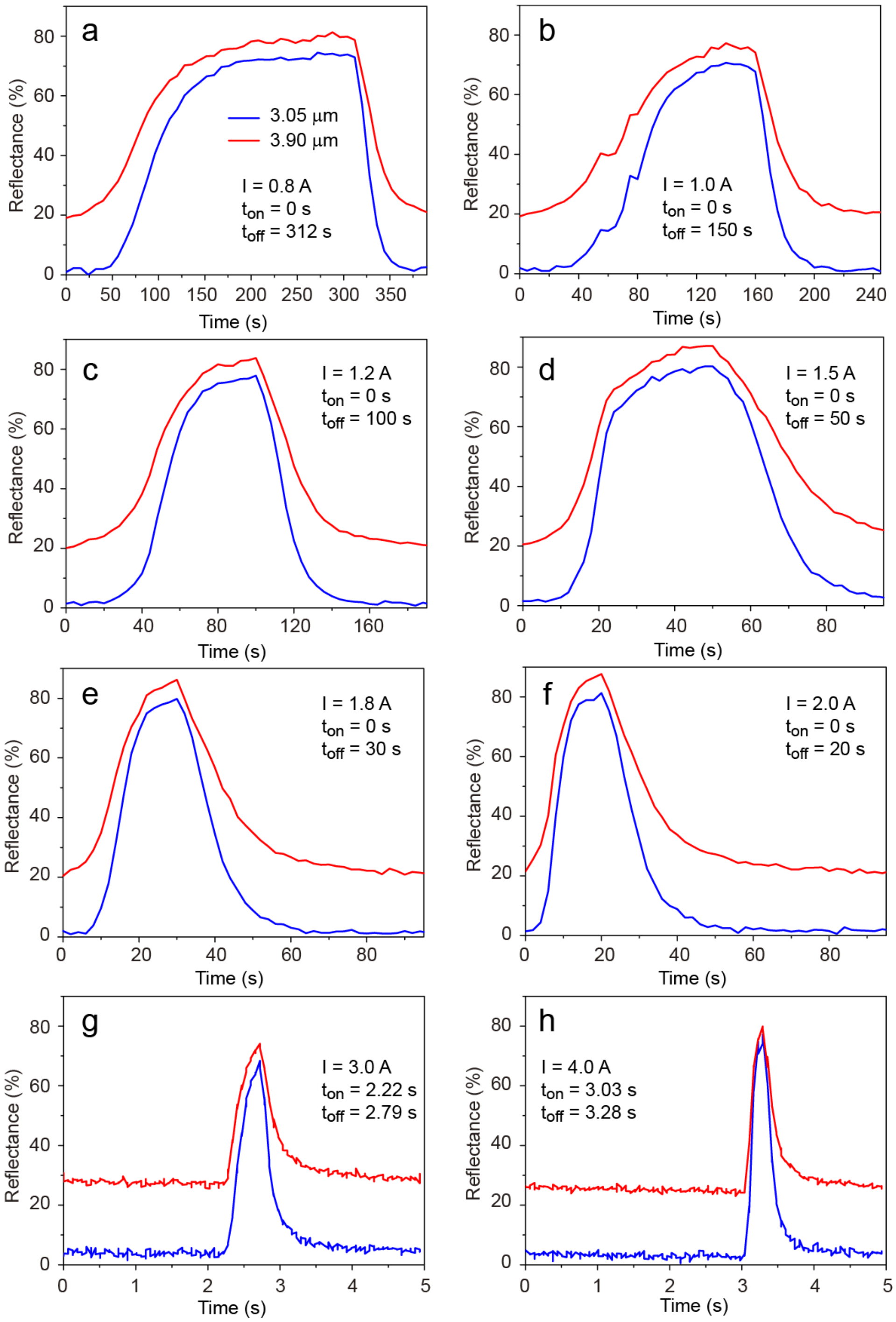

Supplementary Figure 4. On-resonance reflectance of the VO_2 hybrid metamaterial as functions of time for different applied current pulses. In the figures, *I* represents the current intensity, t_{on} is the time when the current pulse is turned on, and t_{off} is the time when the current pulse is turned off.

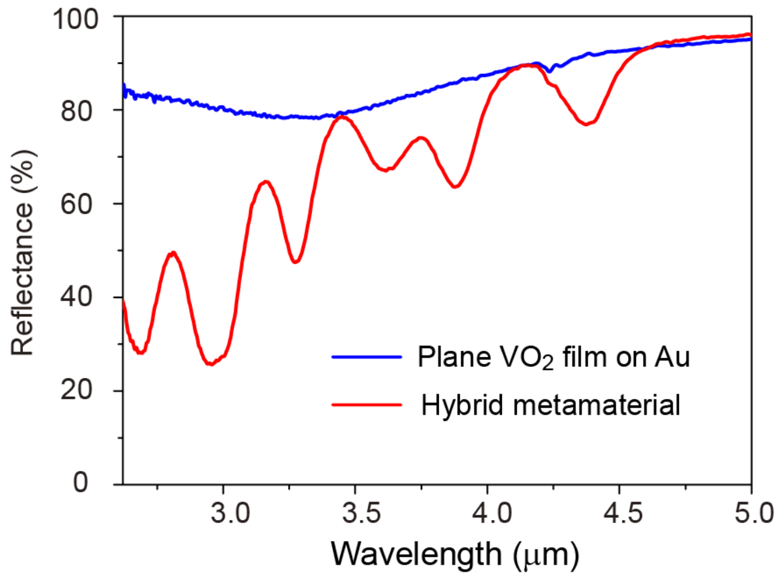

Supplementary Figure 5. Measured reflectance spectra of a VO_2 film on Au and the hybrid metamaterial collected with the FTIR imaging microscope at room temperature. The reflectance spectrum of the hybrid metamaterial indicates significant deviation from that shown in Fig. 2a because of the large angle of light incidence and collection. The images presented in Fig. 4 were accordingly acquired at the resonance around the wavelength of 2.67 μm.

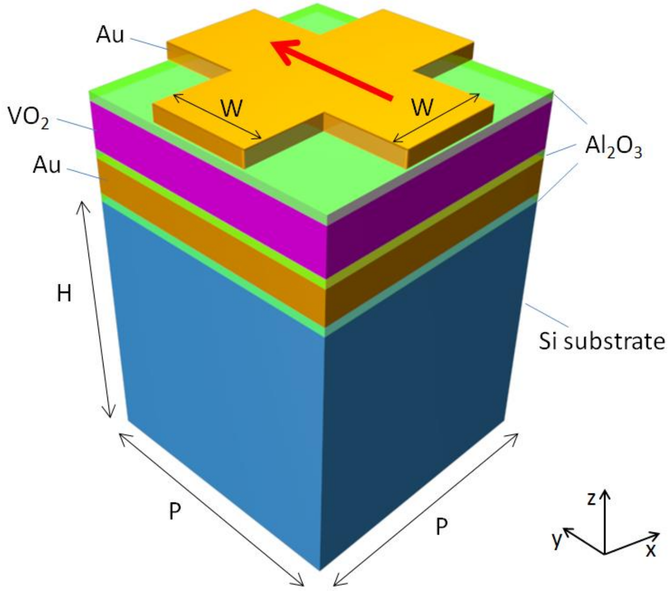

Supplementary Figure 6. Schematic illustration (not drawn to scale) of the structure used for electrical-thermal simulations. Geometrical parameters: H = 500 μm with the others being the same as those used in Fig.1.

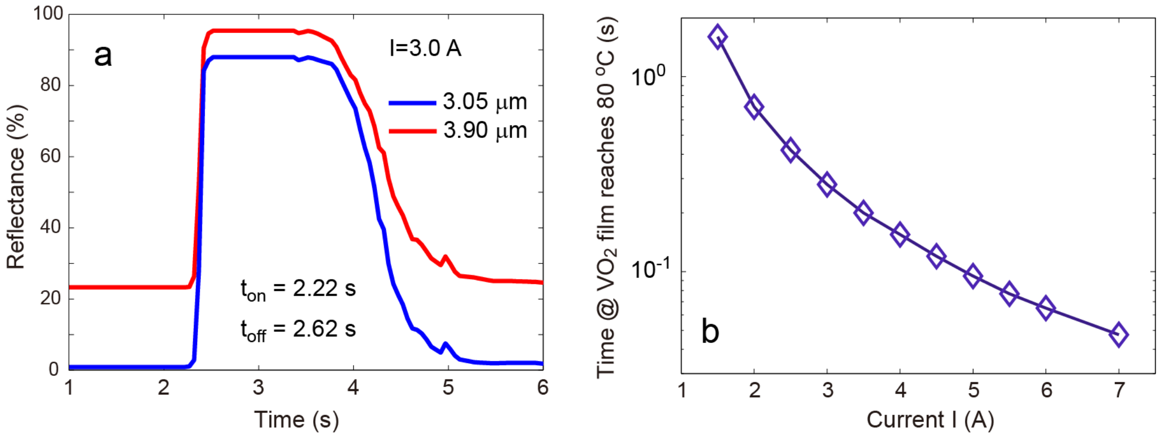

Supplementary Figure 7. Simulated temporal performance of the metadevice. **a**, Simulated on-resonance time-dependent reflectance of the VO_2 hybrid for applied current pulses. **b**, Time for VO_2 film to reach 80 °C as a function of the applied current intensity (t_{on} = 0 s).

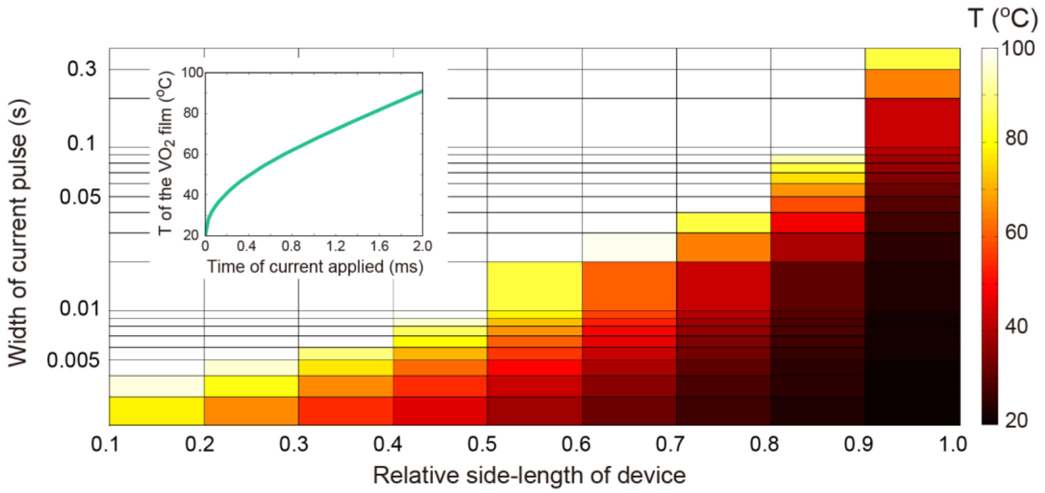

Supplementary Figure 8. Dependence of VO_2 temperature on the dimensions of the metadevice and applied electrical current duration. The side-length was normalized to that of the fabricated metadevice (5×5 mm^2). The inset shows the time-dependent temperature evolution in the VO_2 film for a relative side-length of 0.1. A constant current intensity of 3A was used for all simulations.

Supplementary Table 1 | Comparison of VO_2 enabled active electromagnetic/optical metadevices							
**Ref**	**Trigger of Transition**	**Triggering Method**	**Structure of Sub-unit**	**Modulation Depth (%)**	**Time Scale of Modulation**	**λ_{res} (μm)**	**Year**
**1**	Thermal	Heating Stage	SRR	~25 / T	NA	150	2008
**2**			SRR	~25 / R	NA	3	2009
**3**			Metal Film/Split	>90 / T	NA	400	2010
**4**			VO_2 Cut-wire	~65 / T	NA	400	2010
**5**			Nanostripe Pair	~20 / T	NA	1	2010
**6**			SRR	~80 / T	NA	300	2011
**7**			Y-shaped Resonator	~40 / R	NA	10	2013
**8**			Grating	~60 / R	NA	2	2015
**9**			Hole Array	>90 / T	NA	400	2015
**10**	Optical	THz Pulsed Laser	SRR	~15 / T	Ps	400	2012
**11**		Pulsed UV-light	Metal Disk	~20 / Ext	S	1	2015
**12**	Electrical	Current Source	SRR	~10 / T	ms	300	2009
**13**			SRR	~10 / T	NA	150	2011

Supplementary Note 1. Fabrication of the hybrid metamaterial absorber.

The fabrication process flow for the VO_2 integrated hybrid metamaterial absorber is illustrated in Fig. s1(a-k). The metamaterial was fabricated on a cleaned silicon substrate (Fig. s1a, p-type, 100 orientation, 1.0-10 $\Omega \cdot$ cm, Addison's Engineering, San Jose, CA). Firstly, a 150 nm-thick layer of SiO_2 was grown on the Si substrate by thermal oxidation (wet) (Fig. s1b) to serve as a diffusion barrier between the silicon substrate and the bottom metal layer of the metamaterial (described in the following step). A 20 nm layer of titanium (Ti) and a 150 nm layer of gold (Au) were then deposited on top of the SiO_2 with e-gun evaporation (Fig. s1c, Semicore E-Gun Thermal Evaporator, Semicore Equipment Inc., Livermore, CA). Moreover, Ti serves as an adhesion layer between SiO_2 and Au. A 50 nm-thick layer of Al_2O_3 was then deposited on top of the 150 nm Au layer by atomic layer deposition (ALD) (Fig. s1d, ALD150LE, Kurt J. Lesker, Jefferson Hills, PA). A layer of vanadium oxide VO_x ($x < 2$) was then deposited on top of the sample using pulsed DC reactive ion sputtering with a vanadium metal target at 5 mTorr in a 2.5 % oxygen and 97.5 % argon atmosphere (Fig. s1e, CMS-18 sputter, Kurt J. Lesker, Jefferson Hills, PA). The VO_x film was further oxidized and crystallized in a rapid thermal annealing furnace at 640 °C for 10 minutes in a 0.1 % oxygen and 99.9 % argon atmosphere to form single phase VO_2 (Fig. s1f). Scanning electron microscopy (SEM) and X-ray diffraction (XRD) were used to characterize the VO_2 film (see Fig. s1i,j) and optimize the conditions. After the single phase VO_2 film was formed, the sample was coated with a second layer of Al_2O_3 by using the same ALD process (Fig. s1g) and then ZEP 520 ebeam resist (Zeon, Tokyo) was spin-coated on top of the sample. E-beam lithography (EBPG5200, Raith America Inc., Troy, NY) was performed to define the inverse pattern of the 5 mm $\times$ 5 mm Au-mesh structure on the resist. A 3 nm Ti and 100 nm Au layer was deposited on the sample using the same evaporator, and finally the metal covering the ebeam resist was removed using a lift-off process in 1165 developer (Shipley, Marlborough, MA) to form the Au mesh structure and the electrical contact pads (Fig. s1h). The final device was imaged with SEM (see Fig. 1d).

Supplementary Note 2. Optical characterization.

The normal reflectance of the hybrid metamaterial was characterized with Fourier transform infrared (FTIR) spectroscopy. A customized accessary (Fig. s2) was employed with the integrated Bruker IFS 66/s spectrometer in the FTIR system to realize the normal reflectance measurement. All measured spectra were normalized to that of a gold mirror.

During the measurements for the results shown in Fig. 2 and Fig. 3, the hybrid metamaterial sample was attached to a microscope slide with two layers of parafilm (Bemis Company, Inc, Oshkosh, WI) placed in between. The parafilm under the 5 mm $\times$ 5 mm absorber area was cut away to create thermal isolation between the Si substrate and the microscope slide. The microscope slide was then mounted on the sample holder in the customized FTIR accessary. To enable electro-optic modulation, the Au mesh layer of the sample was connected by external circuitry to a function generator (Agilent 33120A, Agilent Technologies, Inc., Wilmington, DE) and an amplifier (Model BOP 100-4M, Kepco, Inc., Flushing, NY) as the power supply. The applied current was controlled by setting the function generator output (a DC current (Fig. 2) and programed current pulse trains (Fig. 3)) and monitored using the build-in Amp meter on the amplifier. During the measurement for Fig. 3, a thermal couple was attached 1 mm above the surface of the sample to monitor the temperature fluctuation during the writing and erasing operation.

During the measurement for Fig. 4, the hybrid metamaterial sample was photographed using a 128×128 focal plane array detector integrated together with the hyperion 3000 microscope in the FTIR system. The objective lens of the microscope was a 15X condenser with an average incidence and collection angle of light ~30° (N.A. = 0.5). The off-normal incidence effect leads to a deviation of the spectroscopic response during the imaging from the results shown in Fig. 2. For electro-optic control of the IR image, the sample was powered by the same electrical components used in the measurement for Fig. 2. The imaging area of the focal plane array was 340 × 340 μm^2, which is smaller than the entire text area of the sample. Therefore, at each experimental condition, six imaging measurements were carried out sequentially to photograph a section of the text area, and the complete IR image of the sample was obtained through post-processing (stitching) of the multiple 2 dimensional imaging data.

Supplementary Note 3. Thermal modulation of the hybrid metamaterial.

The thermal tuning of the hybrid metamaterial was also tested by directly controlling the temperature of the sample with an external heating element, and the results are shown in Fig. s3. The measurement setup for Fig. s3 was the same as described for the measurement of Fig. 2a except the metamaterial was not connected to any external electrical circuitry, but was instead mounted on a thermoelectric peltier heater (TEC1-12706, Hebei I. T. (Shanghai) Co., Ltd., Shanghai, China). The peltier heater was fixed on the sample holder of the FTIR accessary. The temperature of the sample was tuned by changing the power input of the peltier heater and the corresponding reflectance of the metamaterial was measured at different temperature values after the sample reached thermal equilibrium.

Supplementary Note 4. Dependence of the switching operation on the intensity of applied electrical current.

The switching speed and contrast of the VO_2 integrated hybrid metamaterial closely depends on the intensity and duration of the applied electrical current (I). The current intensity controls the Joule heating power and therefore the temperature rise of the VO_2 film. The duration of the current pulse controls the total power applied to the device and the actual temperature of the film. On the other hand, the thermal energy of the heated VO_2 film simultaneously dissipates to the adjacent environment, including the silicon substrate, surrounding atmosphere, microscope slide used for mounting, and the sample holder in the FTIR accessary. Moreover, this heat dissipation is obviously temperature gradient dependent. Therefore, by utilizing an applied current pulse of larger magnitude and shorter duration, we were able to achieve a more localized Joule heating effect. On the contrary, a global Joule heating, *i.e.*, the temperature of adjacent environment rises significantly during the electro-optic modulation, would result in the requirement of higher total input power for IMT of the VO_2 film and a correspondingly slower time response of the metadevice.

By applying different current pulses, we experimentally studied the switching operation at the two resonances as functions of time and illustrate the measured results in Fig. s4. The resistance of the Au mesh of the sample used is $R = 1\,\Omega$, and the Joule heating power can then be simply expressed as $P = I^2R$. Accordingly, from Fig. s4(a-h), the Joule heating power was 0.64 W, 1 W, 1.44 W, 2.25 W, 3.24 W, 4 W, 9 W and 16 W, respectively. It can be seen that, when the applied power is increased, the "on" and "off" switching time decreases significantly. Fig. s4(a-f) were obtained by sequentially measuring 50 reflectance spectra of the metadevice during the heating-cooling cycle. Whereas, for the

measurement with I of 3.0 A and 4.0 A (Fig. s4(g,h)), we found that the minimum time resolution (~0.12 s) available in the FTIR system is not fine enough to determine the electro-optic time response of the metadevice. To overcome this, we measured the response of 5 pulses and superimposed these measurements into one 5 s window, effectively generating a finer time step and thus a better resolved response curve, as shown in Fig. s4(g,h) with parameters of the current pulses labeled accordingly.

Supplementary Note 5. Electrical-thermal simulations of Joule heating in VO_2 metamaterial absorber.

COMSOL multiphysics software package was used to simulate the Joule heating effect in the VO_2 metamaterial absorber. The purpose of the simulation was to understand the transient thermal behavior of the VO_2 metamaterial absorber. Although rigorous thermal simulation accounting for the complex experimental thermal condition is theoretically possible, it requires computational resources that are currently not easily available. Fortunately, more efficient thermal simulation can be done by making certain assumptions and applying boundary conditions to simplify the experimental condition and, the results should be able to provide useful qualitative insights into the potential ways to improve the switching speed of our electrified hybrid metadevice of this kind.

The geometry of the model is schematically shown in Fig. s6. One unit cell of the VO_2 metamaterial absorber (same as shown in inset 1a) was attached to a silicon substrate of thickness H = 500 μm with a 50nm-thick Alumina layer in between. The Joule heating effect was simulated by coupling an "Electric Currents (ec)" physics model to a "Heat Transfer in Solid (ht)" physics model. Material properties used in this simulation for alumina, silicon and gold were taken from default textbook values.[14] The heat capacity, thermal conductivity, and density of VO_2 were set to be 706.25 J $(kg\cdot K)^{-1}$, 6.7 W $(m\cdot K)^{-1}$, and 4571 kg m^{-3}.[15,16]

The "Electric currents (ec)" physics model was applied to the Au mesh. All boundaries of the Au mesh were set to be electrically insulating except for two boundaries described as follows. The center of the Au mesh was set to the coordinate (0,0,0). The boundary at $y = -P/2$ was assigned a normal current density $J_n = I/A_0$, such that I is the total current applied to the device and $A_0 = w \times t_{Au} \times N$ where t_{Au} and N are the mesh thickness and the number of unit cell along one side-length, respectively. Finally, the boundary at $y = P/2$ was electrically grounded. The red arrow in Fig. s6 indicates the direction of electrical current flow.

The "Heat Transfer in Solid (ht)" physics was applied to all domains and we assumed no net heat flux in the x-y (transverse) plane. The point of interest is set at the geometrical center of the VO_2 layer. Heat transfer in or out of the simulated structure was prohibited by applying a thermal insulation boundary condition to all side-walls at $x = \pm P/2$ and $y = \pm P/2$. All top surfaces were given a convective cooling boundary condition with ambient temperature set to T_0 = 293.15 K and the heat transfer coefficient was set to 5 W m^{-2} K^{-1}. The bottom surface was assigned a thermal dissipation rate of $q_0 = k_{Si}(T_{Si} - T_0)R_{area}$, where k_{Si} is the thermal conductivity of Si, $T_{Si}$is the transient temperature of Si near the bottom surface, and R_{area} is the area ratio of the Si substrate to VO_2 metamaterial absorber in the transverse plain, which is set to 3.25 to accommodate the fact that, in our experiments, the area of the substrate is much larger than that of the metamaterial. This boundary condition is set up to empirically mimic the complex experimental condition (the silicon substrate was attached to a large glass slide and then partially mounted on an FTIR sample holder), and suggests that the bottom surface of Si can

dissipate heat to an infinite heat capacitor at ambient temperature at constant thermal conduction rate (silicon), in a convective cooling fashion.

Fig. s7a illustrates the applied current pulses modulated on-resonance reflectance of the device. Although thermal parameters utilized for all the materials were temperature independent, a good agreement between Fig. s7a and Fig. s4g illustrating the measured data of a similar process clearly indicates the validation of our electrical-thermal simulation. We note that the reflectance evolution in Fig. s7a was obtained by correlating the simulated temperature and the experimental reflectance-temperature data shown in Fig. s2. As we discussed in Fig. s4 and Supplementary Note 4, the switching behavior of the metadevice closely depends on the intensity and duration of the applied electrical current. To better understand the electrical-thermal process, we simulated the temperature-time response of the device and extracted the critical condition of IMT as a function of the current intensity. As illustrated in Fig. s7b, it is obvious that the dynamic response of the device can be dramatically improved by increasing the applied current intensity, which causes a corresponding increase in localized Joule-heating.

On the other hand, the large dimensions (5×5 mm^2) of the sample used, which were favorable for characterization, may not be necessary in practical applications. Metadevices with smaller functional area may require applied current pulses of less intensity and duration, which can facilitate faster modulation at a low level of input power. Figure s8 illustrates the VO_2 temperature as a function of the device dimension and the duration of a 3A current pulse. Temperature higher than 100 °C was left in white color. As is immediately evident in the plot, shrinking the metadevice size can significantly improve its dynamic response, which can be attributed to the reduction of the device's volumetric heat capacity.

Supplementary Note 6. An analysis of the reported VO_2 based metadevices.

It is worth providing an overview of the progress of VO_2 based active metadevices by analyzing the corresponding studies reported to date in the literature. In doing so, we first list the detailed performance comparison of the reported works (Ref. s1 – s13) in Supplementary Table 1 and, below we briefly summarize these studies from three perspectives.

• *Transition triggering method*. As listed in Supplementary Table 1, to the best of our knowledge, three types of transition triggers, *e.g.* thermal, optical and electrical, have been utilized to realize active control of electromagnetic/optical response of VO_2 based metadevices[1-13]. It can be seen that, to trigger the phase transition, most reported works[1-9] employed a straightforward heating stage to provide the device temperature control. Optical excitations have also been used to trigger the transition[10,11]. To date, there are just two demonstrations of an electrically induced transition[12,13] and both operate at THz frequencies (1 - 2 THz).

• *Temporal response*. It is not surprising that no data is available for studies of device functionality based upon the use of a heating stage due to a reliance on global temperature changes, an intrinsically slow process. Besides the study focusing on spatial gradient index[13], the only other work reports (see Ref. s12) a memory effect around 1.0 THz in a hybrid metamaterial with a switching time scale close to 50 milliseconds. The device based on an optically driven transition[10] indicates the potential of the VO_2 integrated metamaterials for ultrafast response modulation.

• *How VO_2 is introduced*. Except for Ref. s4 and s5, all studies listed in Supplementary Table 1 introduced VO_2 into the hybrid systems as a part of the substrate rather than as part of the resonating structure. This design strategy, to some extent, limits the potential modulation depth and temporal response characteristics of the metadevice. Ref. s4 investigated the modulation behavior of resonators directly made of VO_2 which exhibit high indices at THz frequencies, while Ref. s5 revealed thermal regulation induced transmission tuning which was relatively moderate due to the high loss of VO_2 in the near-IR.

Supplementary References:

1. Driscoll, T. *et al.* Dynamic tuning of an infrared hybrid-metamaterial resonance using vanadium dioxide. *Appl. Phys. Lett.* **93**, 024101 (2008).
2. Dicken, M. J. *et al.* Frequency tunable near-infrared metamaterials based on VO_2 phase transition. *Opt. Express* **17**, 18330-18339 (2009).
3. Seo, M. *et al*. Active terahertz nanoantennas based on VO_2 phase transition. *Nano Lett*. **10**, 2064–2068 (2010).
4. Wen, Q. *et al*. Terahertz metamaterials with VO_2 cut-wires for thermal tunability. *Appl. Phys. Lett.* **97**, 021111 (2010).
5. Huang, W. *et al.* Optical switching of a metamaterial by temperature controlling. *Appl. Phys. Lett.* **96**, 261908 (2010).
6. Zhu, J. *et al.* Thermal broadband tunable terahertz metamaterials. *Opt. Commun.* **284**, 3129-3133 (2011).
7. Kats, M. A. *et al.* Thermal tuning of mid-infrared plasmonic antenna arrays using a phase change material. *Opt. Lett.* **38**, 368-370 (2013).
8. Kocer, H. *et al.* Thermal tuning of infrared absorbers based on hybrid gold-VO_2 nanostructures. *Appl. Phys. Lett.* **106**, 161104 (2015).
9. Jeong, Y. *et al.* A vanadium dioxide metamaterial disengaged from insulator-to-metal transition. *Nano Lett*. **15**, 6318–6323 (2015).
10. Liu, M. *et al.* Terahertz-field-induced insulator-to-metal transition in vanadium dioxide metamaterial. *Nature* **487**, 345-348 (2012).
11. Lei, D. Y. *et al.* Optically-triggered nanoscale memory effect in a hybrid plasmonic-phase changing nanostructure. *ACS Photon.* **2**, 1306-1313 (2015).
12. Driscoll, T. *et al.* Memory metamaterials. *Science* **325**, 1518−1521 (2008).
13. Goldflam, M. D. *et al.* Reconfigurable gradient index using VO_2 memory metamaterials. *Appl. Phys. Lett.* **99** 044103 (2011).
14. Haynes, W. M. *CRC handbook of chemistry and physics*. (CRC Press, 2014).
15. Berglund, C. N., Guggenheim, H. J. Electronic properties of VO_2 near the semiconductor-metal transition. *Rhys. Rev.* **185**, 1022-1033 (1969).
16. Gopalakrishnan, G., Ruzmetov, D. & Ramananthan, S. On the triggering mechanism for the metal-insulator transition in thin film VO_2 devices: Electric field versus thermal effects. *J. Mater. Sci.* **19**, 5345-5353 (2009).
